# Intratumoral delivery of mRNA encoding the endogenous TLR2/6 agonist UNE-C1 induces immunogenic cell death and enhances antitumor activity

**DOI:** 10.3389/fimmu.2024.1454504

**Published:** 2024-11-28

**Authors:** Uijoo Kim, Sunkyo Hwang, Seongmin Cho, Hyeong Yun Kim, Hamin Ban, Joohee Park, Jeongwon Mun, Nayoung Kim, Ji Hun Suh, Jihye Choi, Yungyeong Shin, Sang Bum Kim, Ina Yoon, Hyuk-Sang Kwon, Sunghoon Kim

**Affiliations:** ^1^ College of Pharmacy, Yonsei University, Incheon, Republic of Korea; ^2^ Institute for Artificial Intelligence and Biomedical Research, Medicinal Bioconvergence Research Center, College of Pharmacy, Yonsei University, Incheon, Republic of Korea; ^3^ College of Pharmacy, Sahmyook University, Seoul, Republic of Korea; ^4^ Yonsei Institute of Pharmaceutical Sciences, College of Pharmacy, Yonsei University, Incheon, Republic of Korea; ^5^ Zymedi Co., Ltd., Incheon, Republic of Korea; ^6^ Interdisciplinary Graduate Program in Integrative Biotechnology, Yonsei University, Incheon, Republic of Korea; ^7^ College of Medicine, Yonsei University, Seoul, Republic of Korea

**Keywords:** cancer immunotherapy, toll-like receptor, intratumoral treatment, mRNA therapeutics, tumor microenvironment, immunogenic cell death

## Abstract

**Introduction:**

Recent investigations have highlighted the intratumoral administration of Toll-like receptor (TLR) ligands as a promising approach to initiate localized immune responses and enhance antitumor immunity. However, the clinical application of these ligands is limited by their rapid dissemination from the tumor microenvironment, raising concerns about reduced effectiveness and systemic toxicity.

**Methods:**

To address these challenges, our study focused on the intratumoral delivery of mRNA encoding UNE-C1, a TLR2/6 ligand known for its efficacy and low toxicity profile. We explored the potential of UNE-C1 to induce immunogenic cell death (ICD) through autocrine mechanisms, facilitated by the release of damage-associated molecular patterns (DAMPs) triggered by TLR2 activation.

**Results:**

Our findings indicate that sensitivity to UNE-C1-induced cell death is dependent on the expression levels of TLR2 and the Fas-associated death domain (FADD) in cancer cells. Furthermore, we investigated the paracrine activation of dendritic cells (DCs) by UNE-C1 via TLR2 signaling, which primes a CD8+ T cell response essential for tumor regression.

**Discussion:**

Our results advocate for the intratumoral delivery of UNE-C1 via mRNA therapy as a promising strategy for innovative antitumor treatments.

## Introduction

Aminoacyl-tRNA synthetases (ARSs) are enzymes that play a pivotal role in protein translation by facilitating the attachment of amino acids to their corresponding transfer RNAs (tRNAs). Beyond their conventional role in protein synthesis, ARSs are involved in various cellular processes, including metabolism, inflammation, angiogenesis, and tumorigenesis (1–5). Evolutionary adaptations, such as the acquisition of additional domains—some common to multiple ARSs and others unique to individual ARSs—have expanded the functional repertoire of these enzymes (6, 7). Notably, the extracellular release of cytosolic ARSs contributes to diverse physiological and pathological roles, including immune modulation (8, 9). For instance, the N-terminal extension domain of tryptophanyl-tRNA synthetase 1 (WARS1) triggers innate immune responses by interacting with the Toll-like receptor (TLR)4-myeloid differentiation factor 2 (MD2) complex in macrophages during pathogenic stimuli (10). Similarly, the secretion of human cysteinyl-tRNA synthetase 1 (CARS1), which harbors the TLR2/6 binding motif known as the UNE-C1 domain, activates an immune response. The use of UNE-C1 as an adjuvant in cancer vaccines has been shown to enhance antitumor responses by boosting tumor-specific immunity (11).

TLRs play a vital role in initiating both innate and adaptive immune responses, thereby triggering anti-cancer immune reactions (12–14). TLR activation occurs upon the recognition of diverse pathogen-associated molecular patterns (PAMPs) and damage-associated molecular patterns (DAMPs), leading to the secretion of proinflammatory cytokines from antigen-presenting cells like dendritic cells (DCs) and macrophages (15). This cascade amplifies the activity of cytotoxic T lymphocytes, fostering robust anti-cancer immune responses. Despite extensive exploration of numerous TLR agonists as potential anti-cancer therapies, only two have gained approval for patient use due to concerns related to cytokine release syndrome and limited efficacy (14, 16–18). Bacillus Calmette-Guérin (BCG), a TLR2/4 agonist, is commonly administered to patients with non-muscle-invasive bladder cancer, and imiquimod, a TLR7 agonist, is used in individuals with superficial basal cell carcinoma (14). BCG therapy can result in complications, with approximately 8% of patients discontinuing treatment due to adverse effects (19). Similarly, imiquimod also triggers various side effects, including skin conditions and systemic manifestations (20). Therefore, developing a TLR agonist with minimal side effects is imperative to improve patient tolerance and treatment outcomes.

Stimulating antitumor immune responses within the tumor microenvironment is an effective strategy to optimize the efficacy of immunotherapy while minimizing systemic toxicity. Directly delivering immune stimulants into tumors facilitates the induction of such localized immune responses. Several ongoing clinical studies are focusing on the intratumoral administration of diverse immune stimulants, including TLR agonists, to enhance anti-cancer responses while reducing toxicity (21–25). The intratumoral delivery of TLR2/3 agonists has been shown to induce ICD in cancer cells, highlighting the potential efficacy of this approach (26). However, challenges persist due to the rapid systemic diffusion of therapeutics within tumors, compromising their effectiveness and leading to systemic proinflammatory reactions (25). To address this challenge, studies are focusing on delivering mRNA encoding immune stimulants, including various cytokines, instead of the immune stimulants themselves (27, 28). This approach allows for sustained intratumoral expression over extended periods, overcoming limitations associated with the rapid systemic diffusion and short half-life of directly administered immune stimulants (29).

UNE-C1 emerges as an ideal candidate for mRNA-based therapies due to its documented low systemic toxicity and impressive efficacy (11). In this study, we utilized UNE-C1, a TLR2/6 agonist, in local mRNA therapy for tumors to demonstrate its ability to activate innate immunity and induce ICD in cancer cells. Moreover, we investigated the cell death induced by UNE-C1 through the TLR2-mediated apoptosis pathway involving the Fas-associated death domain (FADD). The findings of our study underscore the potential of UNE-C1 as a potent standalone anticancer agent.

## Materials and methods

### Cell culture

MCA205, AsPC-1, Panc10.05, and HT-1080 cells were cultured in RPMI-1640 medium (Cytiva, SH30255.01) supplemented with 10% fetal bovine serum (FBS) (Cytiva, SH30084.03) and 1% penicillin-streptomycin (Cytiva, SV30010). MC38 and Caco-2 cells were cultured in DMEM medium (Cytiva, SH30243.01) supplemented with 10% FBS and 1% penicillin-streptomycin. These cells were maintained in a humidified atmosphere with 5% CO_2_ at 37°C.

### Cell viability and cytotoxicity assays

To assess cell viability, cells were seeded at a density of 5 × 10^3^ cells per well in 96-well plates and incubated overnight. Subsequently, they were treated individually with recombinant UNE-C1 (0.1, 0.5 and 1 μM), MTX (2 μM), and 0.5 μM of proteinase K digested UNE-C1 for 24 h. To investigate the involvement of various cell death pathways, some cells were pre-incubated individually with cell death inhibitors: 10 μM Z-DEVD-FMK, 1 μM ferrostatin-1, and 20 μM necrostatin-1, for 12 h before being treated individually with recombinant UNE-C1 (0.5 μM). Cell viability was assessed using the Cell Counting Kit-8 (CCK-8) assay (Dojindo Laboratories, CK04) following the manufacturer’s instructions.

To assess cytotoxicity, cells were seeded at a density of 5 × 10^4^ cells per well in 24-well plates and subsequently transfected with siRNA (10 pmol). Following a 24 h incubation, cells were treated individually with recombinant UNE-C1 (0.5 μM) and MTX (2 μM) for 24 h. Cytotoxicity was assessed using the LDH Assay Kit-WST (Dojindo Molecular Technologies, CK12) following the manufacturer’s instructions.

### ATP release assay

Cells were seeded at a density of 5 × 10^4^ cells per well in 24-well plates and treated individually with recombinant UNE-C1 (0.1, 0.5 and 1 μM) and MTX (2 μM) for 1 h. In another experiment, UNE-C1 and MTX treatments were administered after TLR2 knockdown. Extracellular ATP release level was measured using the ATP Assay Kit (Promega, G7570) following the manufacturer’s instructions.

### Detection of calreticulin exposure

Cells were seeded at a density of 5 × 10^4^ cells per well in 24-well plates and treated individually with recombinant UNE-C1 (0.1, 0.5 and 1μM) and MTX (2 μM) for 18 h. In another experiment, the UNE-C1 and MTX treatments were applied after TLR2 knockdown. After treatment, cells were detached using 5 mM EDTA in PBS and then centrifuged at 500 × *g* for 10 min at 4 °C. Then, the cells were washed with fluorescence-activated cell sorting (FACS) buffer (1% BSA in PBS) and incubated on ice for 30 min in FACS buffer containing propidium iodide (PI) (BioLegend, 421301) (1:50) and anti-calreticulin antibody (Abcam, ab196158) (1:100). The stained cells were analyzed using accuri C6 flow cytometer (Becton Dickinson, USA).

### Detection of HMGB1 release

Cells were seeded at a density of 1 × 10^6^ cells in 6-well plates and transfected with siRNA (30 pmol). After 24 h incubation, the cells were treated individually with recombinant UNE-C1 (0.1, 0.5 and 1 μM) and MTX (2 μM) for 24 h. The culture supernatants were collected by centrifuging at 500 × *g* for 10 min and further centrifuged at 10,000 × *g* for 30 min to remove residual debris. Protein precipitation was achieved by adding trichloroacetic acid (TCA) (Sigma-Aldrich, 76-03-9) to the supernatant to obtain a final concentration of 12%, followed by overnight incubation at 4°C. Subsequently, the samples were centrifuged at 18,000 × *g* for 15 min, and the resulting supernatant was neutralized using 0.1 M 4-(2-hydroxyethyl)-1-piperazineethanesulfonic acid (HEPES; pH 8.0). Then the samples were separated using 10% SDS-PAGE and transferred onto a PVDF membrane for immunoblotting with an anti-HMGB1 antibody (Abcam, ab18256).

### Evaluation of TLR2 expression

Flow cytometry was used to quantify TLR2 expression levels in various cell lines, and to confirm the reduction in expression levels following TLR2 knockdown. The cell lines were stained with an anti-TLR2 antibody (BioLegend, 153003) (1:100) as described above.

### *In vitro* bioactivity assays for mRNA encoding UNE-C1

MCA205 cells were seeded at a density of 2.5 × 10^5^ in 6-well plates, transfected with 5 μg of mRNA encoding either UNE-C1 or luciferase using Lipofectamine MessengerMAX (Thermo Fisher Scientific, LMRNA008), and incubated for 24 h. Subsequently, the samples were centrifuged at 500 × *g* for 10 min at 4 °C. The resulting supernatants were used to measure ATP release levels, while the pelleted cells were used to determine the percentage of PI^+^ cells and assess calreticulin exposure. To detect secreted UNE-C1 in the culture medium, protein precipitation was performed by adding TCA to the supernatant as previously described.

Mouse bone marrow-derived DCs (BMDCs) were seeded at density of 5 × 10^5^ cells in 24-well plates. These cells were treated with 150 μl of supernatant harvested from MCA205 cell cultures 24 h after mRNA transfection. After a 24 h incubation, the cells were harvested and stained with antibodies specific for CD11c (BioLegend, 117390), CD80 (BioLegend, 104721) and CD86 (BioLegend, 105005). The stained cells were then analyzed using flow cytometry.

### Western blotting

The cells were lysed with lysis buffer (25 mM Tris-HCl (pH 7.4), 1% Triton X-100, 150 mM NaCl, 2 mM EDTA, 10% glycerin, 0.1% SDS, and protease inhibitor) for 30 min at 4 °C. Then, the lysates were centrifuged at 13,500 × *g* for 15 min at 4 °C to remove debris. Protein concentrations in the supernatants were quantified using the Pierce BCA Protein Assay Kit (Thermo Fisher Scientific, 23227). The supernatants were diluted with 5X SDS sample loading buffer (250 mM Tris-HCl (pH 6.8), 50% glycerol, 3.575 M 2-mercaptoethanol, and 10% SDS) and the resulting samples were boiled for 15 min at 100 °C. Equal amounts of protein were subjected to SDS-PAGE and transferred onto PVDF membranes. Subsequently, the membranes were blocked with TBS-T supplemented with 0.1% Tween 20 (VWR, 0777-1L) and 5% skim milk for 1 h. Then, the membranes were incubated overnight at 4 °C with primary antibodies specific to HMGB1 (Abcam, ab18256), CARS1 (In-house), FADD (Cell Signaling, 2782), caspase-3 (Cell Signaling, 9662), FAS (Cell Signaling, 4233), BCL-2 (MilliporeSigma, 04-436), TRADD (Cell Signaling, 3684), caspase-8 (Cell Signaling, 9746), TRAF2 (Santa Cruz, sc-876) and β-actin (Santa Cruz, sc-47778). Next, the membranes were probed with cognate HRP-conjugated IgG secondary antibodies. Protein bands were visualized using ECL solutions (AbClon, ABC-3001) and relative intensities were analyzed using ImageJ.

### mRNA synthesis

The mRNA sequences, including 5′ UTR, 3′ UTR, and signal peptides, were derived from a previous study (30). DNA templates were synthesized via PCR amplification of the corresponding plasmids using a forward primer and a reverse primer containing a 120T sequence at the 5′ end. mRNA was synthesized using the HiScribe T7 High Yield RNA Synthesis Kit (NEB, E2040) in the presence of N1-methyl-pseudouridine (TriLink, N-1081) and co-transcriptionally capped using CleanCap AG (TriLink, N-7113). Subsequently, the samples were treated with DNase I (NEB, M0303S) to remove residual DNA, and mRNA was purified using the Monarch RNA Cleanup Kit (NEB, T2040L).

### Lipid nanoparticle formulation

LNPs were formulated by mixing an aqueous phase containing the mRNA with an ethanol phase containing the lipids through pipetting. The ethanol phase was prepared by solubilizing D-Lin-MC3-DMA (MCE, HY-112251), DSPC (Sigma, P1138), cholesterol (Sigma, C3045), and DMG-PEG 2000 (Avanti, 880151P) in ethanol at a molar ratio of 50:10:38.5:1.5. The aqueous phase was prepared by dissolving the mRNA in 50 mM sodium acetate (pH 4.0). This aqueous phase was then mixed with the ethanol phase, which contained the lipids, at a molar ratio to achieve an ionizable lipid/mRNA weight ratio of 10:1 through repeated pipetting.

### Transfection

Lipofectamine RNAiMAX (Thermo Fisher Scientific, 13778-075) was used to transfect siRNAs following the manufacturer’s instructions. Briefly, a mixture of 5 pmol siRNA and 25 µL OptiMem was combined with a mixture of 1.5 µL RNAiMAX and 25 µL OptiMem. The combined mixture was mixed gently, incubated for 5min at room temperature, and then added dropwise into each well of 24-well plates. After 24 h, further experiments were conducted.

For mRNA transfection, Lipofectamine MessengerMAX Transfection Reagent (Thermo Fisher Scientific, LMRNA008) was used. Cells were seeded at a density of 2.5 × 10^5^ cells in 6-well plates. A mixture of 2.5 µg mRNA and 3.75 µL MessengerMAX reagent in 250 µL OptiMem was prepared. After a 5 min incubation at room temperature, the mRNA reagent mixture was added to the cells. After 4 h, the medium of transfected cells was replaced with a serum-free medium, and the cells were incubated for an additional 24 or 48 h.

### Immunohistochemistry

Tumor tissues were resected from mice 3 d post-treatment. The tissues were then fixed in 4% paraformaldehyde, embedded in paraffin wax, and sectioned (5 μm) onto microscope slides. The sections were rehydrated in xylene and boiled in citric acid buffer (10 mM, pH 6.0) for antigen retrieval. Subsequently, they were incubated with 0.3% hydrogen peroxide and then blocked with goat serum. Then, the sections were incubated overnight at 4 °C with primary antibodies: anti-HMGB1 (Abcam, ab18256), anti-Calreticulin (Abcam, ab2907), anti-CD8 (Abcam, ab203035), and anti-cleaved caspase-3 (Cell Signaling, 9661L). After mounting, sections were imaged using Pannoramic MIDI slide scanners (3DHISTECH). Representative images for each section were captured at 40x magnification and analyzed using FIJI (Image-J-based open-source software).

### Quantitative real-time PCR

Tumor tissues were resected 24 and 72 h post-treatment and homogenized using BioMasherII (BioMasher, 890864). Total RNA from tumor homogenates was isolated using the RNeasy Mini Kit (Qiagen, 74106). cDNA was synthesized using the Maxima cDNA synthesis kit (Thermo Fisher Scientific, K1642). qRT-PCR was performed using the TB Green Premix (Takara, RR820A) and a Thermal Cycler Dice Real-Time System III (Takara, TP950). Reactions were performed in triplicate, and the threshold cycle numbers were averaged. Gene expression levels were normalized to the housekeeping gene mouse GAPDH. All primer sequences used in qRT-PCR are shown in **Table 1**.

**Table 1:** Sequence of primers.

Gene	Primer sequence	Product size (bp)
*IFN-γ*	F : TGGCTGTTTCTGGCTGTTAC R : TCCACATCTATGCCACTTGAGTT	91
*Granzyme B*	F : GACAACACTCTTGACGCTGG R : TGATCTCCCCTGCCTTTGTCC	108
*Perforin*	F : GATGTGAACCCTAGGCCAGA R : GGTTTTTGTACCAGGCGAAA	161
*GAPDH*	F : CTCCCACTCTTCCACCTTCG R : GCCTCTCTTGCTCAGTGTCC	189

### *In vivo* tumor model

MCA205 cells (5 × 10^5^) were injected subcutaneously into the right flank of both C57BL/6 and athymic nude mice. When tumor volumes reached 50 mm^3^, the mice were injected with either 5 μg of LNP, 5 μg of recombinant UNE-C1, or 10 mg/kg of doxorubicin as indicated. Tumor sizes were measured with a digital caliper and calculated using the formula (length × width^2^) × 0.5. Tumor-draining lymph nodes (TdLN) were harvested 3 d after intratumoral treatment. The lymph nodes were passed through a 70 μm strainer, and red blood cells were removed. Single cells were stained with antibodies against CD11c (BioLegend, 117310), CD8 (BioLegend, 100706), CD103 (BioLegend, 121405), XCR1 (BioLegend, 148207), CD172a (BioLegend, 144011), CD11b (BioLegend, 101229), F4/80 (BioLegend, 123107), MHCII (BioLegend, 107607), CD206 (BioLegend, 141707), and B220 (BioLegend, 103234) to identify CD8^+^ DCs (CD11c^+^, CD8α^+^, and B220^-^), CD103 DCs (CD11c^+^, CD103^+^, and B220^-^), DC2 (CD11c^+^, XCR1^-^, and CD172a^+^), TAM1 (CD11b^+^, F4/80^+^, CD206^-^, and MHCII^+^) and TAM2 (CD11b^+^, F4/80^+^, CD206^+^, and MHCII^-^) as described previously. The cytometry plots were provided in the **Supplementary Figures**.

### Analysis of tumor-infiltrating immune cells

Tumor cells from mice were dissociated using a tumor dissociation kit (Miltenyi Biotec) following the manufacturer’s protocol. After red blood cell lysis, tumor cells were stained with antibodies against CD8 (BioLegend, 100733), CD3 (BioLegend, 100236), CD11c (BioLegend, 117310), CD103 (BioLegend, 121405), MHCII (BioLegend, 107605), and B220 (BioLegend, 103234) to identify CD8^+^ T cells (CD3^+^ and CD8^+^) and CD103^+^ DCs (CD11c^+^, MHCII^+^, B220^-^, and CD103^+^) as described previously. For intracellular staining of IFN-γ, perforin, and Granzyme B, tumor-infiltrating immune cells were incubated with PMA (Sigma, P8193), ionomycin (Sigma, I0634) and BFA (BD, 51-2301KZ) for 4 h. Fixation and permeabilization solutions (BD, 554714) were employed to facilitate the staining of intracellular IFN-γ (BioLegend, 505808), perforin (BioLegend, 154305), and Granzyme B (BioLegend, 372207). The frequencies of IFN-γ^+^, perforin^+^, and Granzyme B^+^ cells were analyzed among CD3^+^ CD8^+^ and CD3^+^ CD4^+^ T cells. Flow cytometry was used to analyze the stained cells. The cytometry plots were provided in the **Supplementary Figures**.

### Statistical analysis

Data were analyzed using GraphPad Prism V.7.0, and results are presented as mean ± SD or SEM. Statistical significance was analyzed using Student’s *t*-tests, with *p* < 0.05 indicating statistically significant differences between two groups.

## Results

### UNE-C1 triggers ICD in cancer cells via the TLR2 pathway

**Figure 1:**
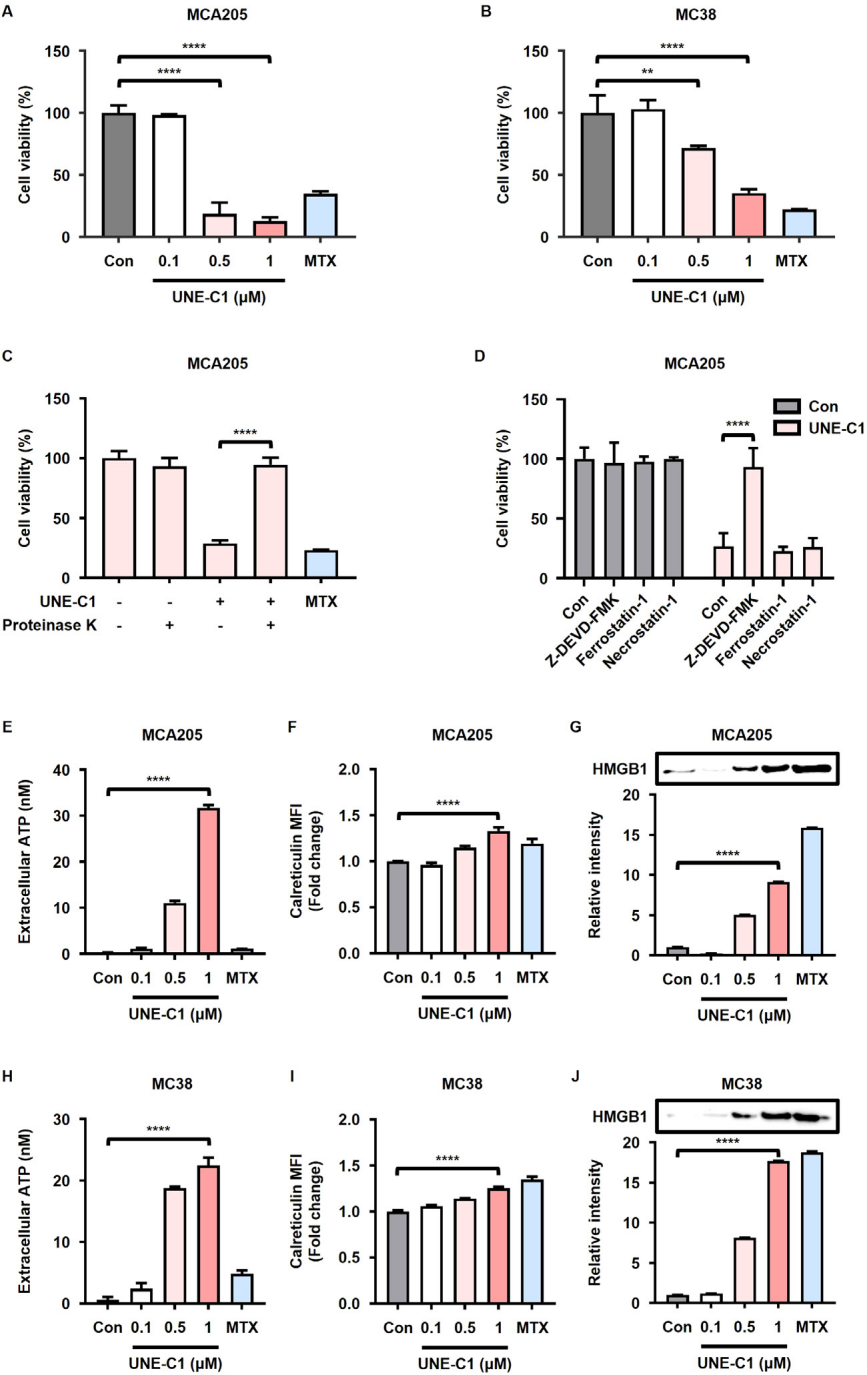
UNE-C1 induces immunogenic cell death (ICD) in MCA205 and MC38 cancer cells. **(A, B)** Cell viability of **(A)** MCA205 and **(B)** MC38 cells treated with UNE-C1 or MTX. **(C, D)** Cell viability of MCA205 cells treated with **(C)** UNE-C1, MTX or UNE-C1 digested with proteinase K, and **(D)** UNE-C1 following pre-incubation with Z-DEVD-FMK, ferrostatin-1, or necrostatin-1. **(E, H)** Extracellular ATP concentrations in **(E)** MCA205 and **(H)** MC38 cells following treatment with UNE-C1 or MTX. **(F, I)** Flow cytometric analyses showing cell-surface calreticulin induction on **(F)** MCA205 and **(I)** MC38 cells after treatment with UNE-C1 or MTX. **(G, J)** Secreted HMGB1 levels in the conditioned media of **(G)** MCA205 and **(J)** MC38 cells after UNE-C1 or MTX treatment. Results are presented as mean ± SD. Statistical significance was determined using one-way ANOVA (**p < 0.01 and ****p < 0.0001).

Given previous findings regarding the induction of ICD by various TLR agonists (26, 31), we investigated whether UNE-C1 could elicit ICD in cancer cells. We treated MCA205 and MC38 cells with UNE-C1 and observed a dose-dependent induction of cancer cell death (**Figures 1A, B**). This effect was directly attributed to the recombinant protein UNE-C1, as the observed activity ceased when UNE-C1 was completely degraded using proteinase K (**Figure 1C**, **Supplementary Figure 1A**). Moreover, the study demonstrated the inhibitory effect of a caspase-3 inhibitor (Z-DEVD-FMK) on UNE-C1-induced cell death, whereas necroptosis and ferroptosis inhibitors (necrostatin-1 and ferrostatin-1, respectively) were ineffective in preventing this cell death (**Figure 1D**). These results suggest the involvement of the apoptosis pathway mediated by active caspase-3. Furthermore, there was an elevation in markers that signify ICD, such as the increased release of ATP and HMGB1, as well as membrane exposure of calreticulin, in UNE-C1-treated apoptotic cells (**Figures 1E–J**). Compared to MTX, a well-established inducer of ICD, UNE-C1 significantly enhanced the ICD markers. Based on these findings, we conclude that UNE-C1 induces ICD in cancer cells.

**Figure 2:**
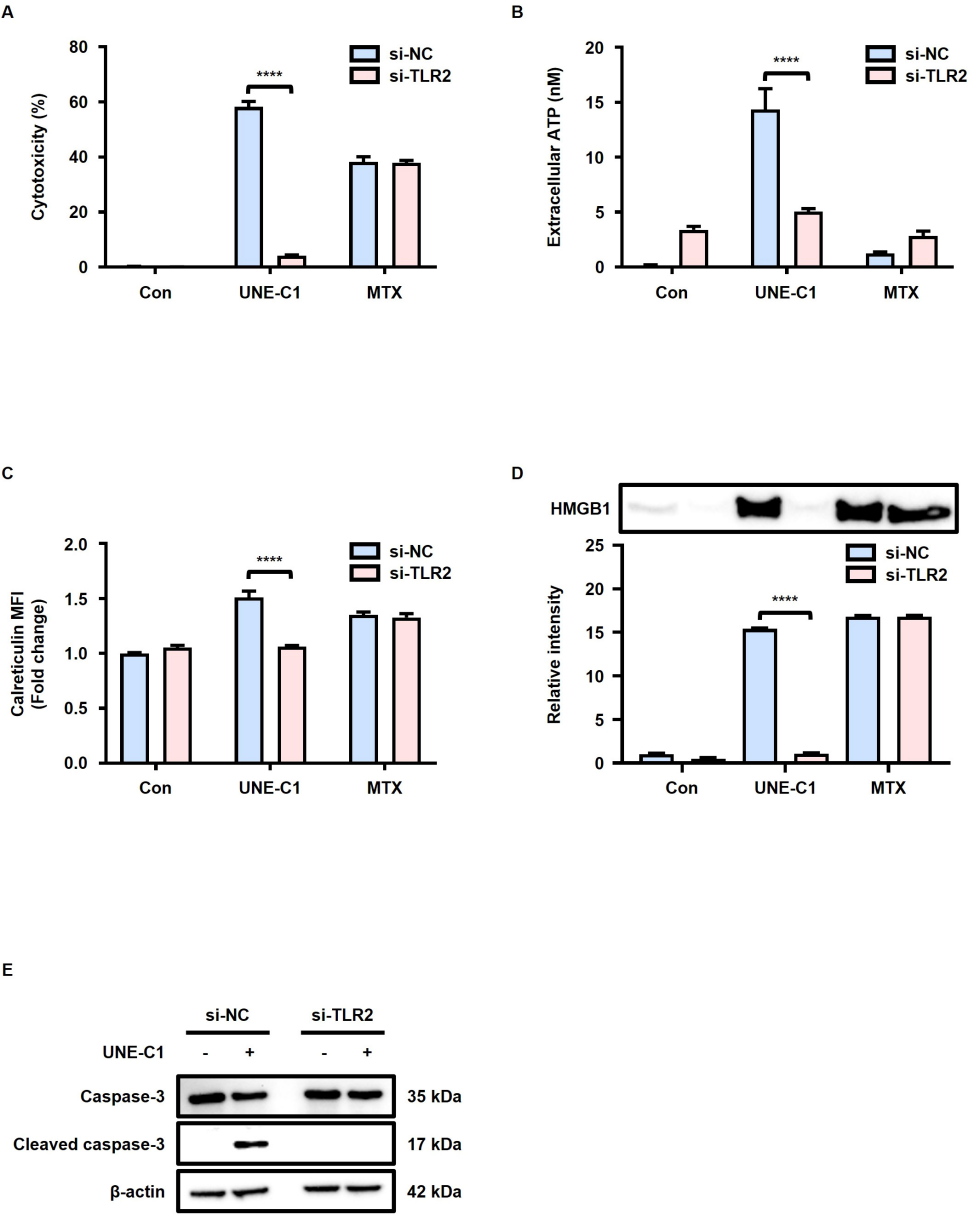
UNE-C1 induces TLR2 dependent ICD. **(A–E)** Evaluation of the effect of TLR2 knockdown in MCA205 cells in response to UNE-C1 or MTX treatment. **(A)** Assessment of cellular viability, **(B)** quantification of extracellular ATP levels, **(C)** determination of calreticulin surface expression, and **(D)** immunoblot analysis of secreted HMGB1 in conditioned media. **(E)** Immunoblot analysis of caspase-3, cleaved caspase-3, and β-actin in MCA205 cells treated with UNE-C1 or MTX post-transfection with either control siRNA (si-NC) or TLR2 siRNA (si-TLR2). Results are presented as mean ± SD. Statistical significance was determined using two-way ANOVA (****p < 0.0001).

To investigate the potential involvement of TLR2 in the induction of ICD by UNE-C1, we conducted experiments using MCA205 cells transfected with siRNA targeting TLR2. The reduction in TLR2 surface expression levels in MCA205 cells transfected with TLR2 siRNA was confirmed (**Supplementary Figure 2A**). While the cytotoxicity of MTX was not affected by TLR2 knockdown, the cytotoxicity of UNE-C1 was reduced in TLR2 knockdown cells compared to cells transfected with control siRNA (**Figure 2A**). These results suggest the involvement of TLR2 in UNE-C1-induced cell death. Furthermore, the release of ATP and HMGB1, as well as calreticulin exposure induced by UNE-C1, was reduced in cells transfected with TLR2 siRNA (**Figures 2B–D**). The activation of caspase-3 by UNE-C1 was inhibited in cells with TLR2 knockdown (**Figure 2E**). These results suggest that UNE-C1 induces cell death by activating caspase-3 through TLR2. Overall, these findings provide compelling evidence that UNE-C1 triggers ICD through TLR2-mediated mechanisms.

### Stimulation of the innate immune system and induction of ICD by mRNA encoding UNE-C1

**Figure 3:**
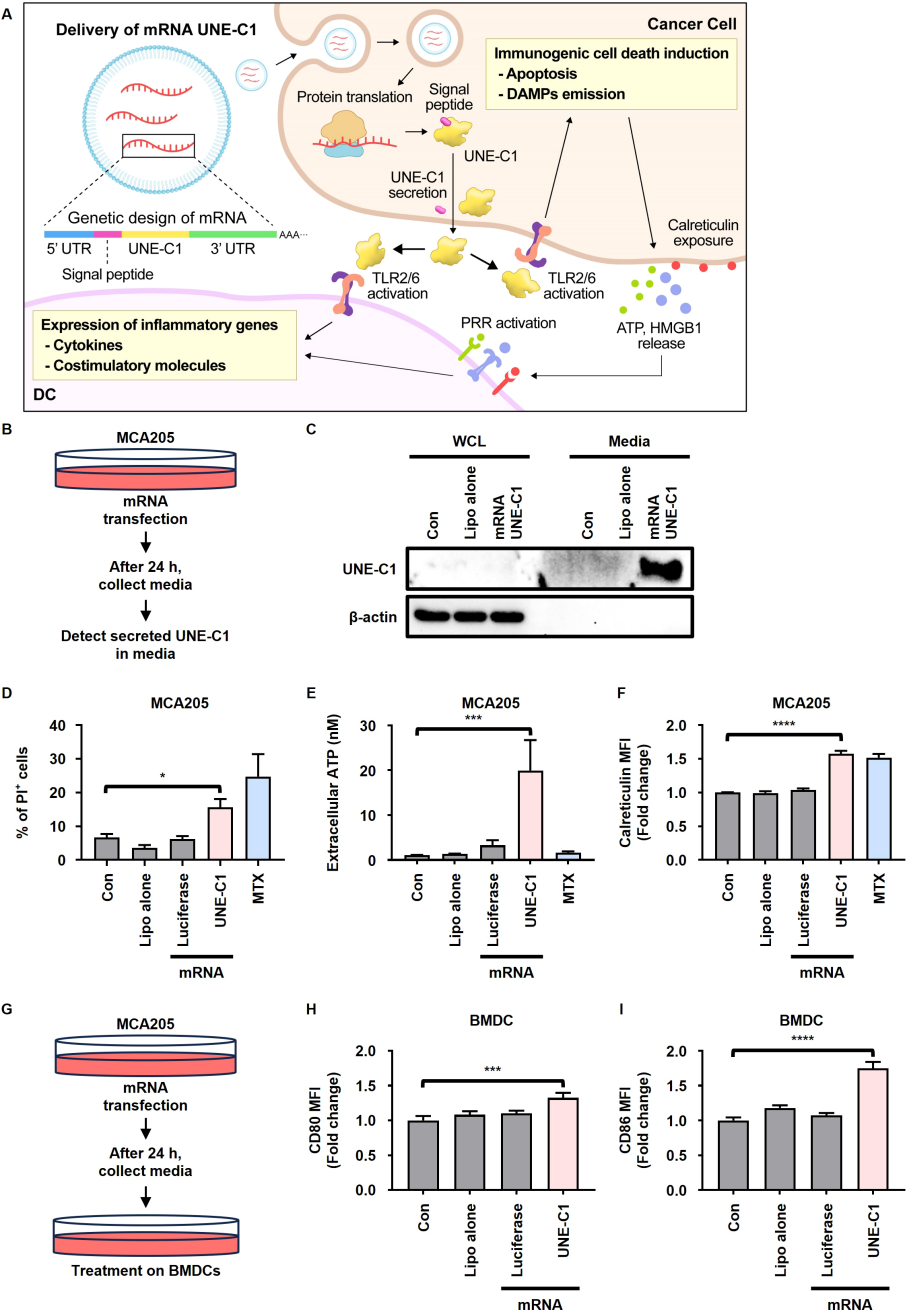
mRNA encoding UNE-C1 induces ICD and activates innate immune cells. **(A)** Schematic representation of the mechanism of action underlying ICD induction and innate immune activation by mRNA UNE-C1. **(B–I)** Investigation of UNE-C1 expression and functional efficacy *in vitro* after mRNA administration. **(B)** Schematic representation detailing the method for detecting UNE-C1 secretion in culture media through protein precipitation, facilitated by mRNA delivery. **(C–F)** Post-transfection with indicated mRNA or lipofectamine alone (Lipo alone) in MCA205 cells, **(C)** immunoblot analysis to identify secreted UNE-C1, **(D)** evaluation of cellular viability through propidium iodide (PI) staining, **(E)** quantification of extracellular ATP levels, and **(F)** analysis of calreticulin surface expression. **(G)** Schematic representation detailing the method for detecting dendritic cell (DC) activation post-treatment with supernatant from MCA205 cells transfected with indicated mRNA. **(H, I)** Flow cytometric analysis of DC activation markers, specifically **(H)** CD80 and **(I)** CD86. Results are presented as mean ± SD. Statistical significance was determined using one-way ANOVA (*p < 0.05, ***p < 0.001, and ****p < 0.0001).

We generated mRNA encoding UNE-C1 conjugated to a signal peptide (mRNA UNE-C1) (**Supplementary Figure 3A**) and assessed whether the expressed UNE-C1 was secreted extracellularly, subsequently promoting ICD and activating innate immune responses (**Figure 3A**). After transfecting MCA205 cells with this mRNA, we found that UNE-C1 was highly expressed in the culture medium (**Figures 3B, C**). The protein expressed by mRNA UNE-C1 was found to have the same molecular size as the recombinant UNE-C1 protein and was secreted at a concentration of approximately 500 nM, which was sufficient to induce ICD in cancer cells (**Supplementary Figure 3B**). The transfected MCA205 cells exhibited cell death accompanied by elevated ATP release and calreticulin exposure (**Figures 3D–F**, **Supplementary Figure 3C**). Additionally, we treated BMDCs with supernatants from MCA205 cells transfected with mRNA, as outlined in the schematic (**Figure 3G**). We then determined the expression levels of costimulatory molecules, CD80 and CD86, which are known activation markers for DCs. Consistently, UNE-C1 derived from mRNA transfection led to the upregulation of these molecules (**Figures 3H, I**). Based on these results, we conclude that delivering UNE-C1 as mRNA induces ICD in cancer cells through an autocrine pathway and activates innate immune cells through a paracrine pathway. Thus, mRNA is an effective delivery platform for UNE-C1.

### Intratumoral delivery of mRNA encoding UNE-C1 induces antitumor activity by recruiting innate immune cells and activating T cell immunity

**Figure 4:**
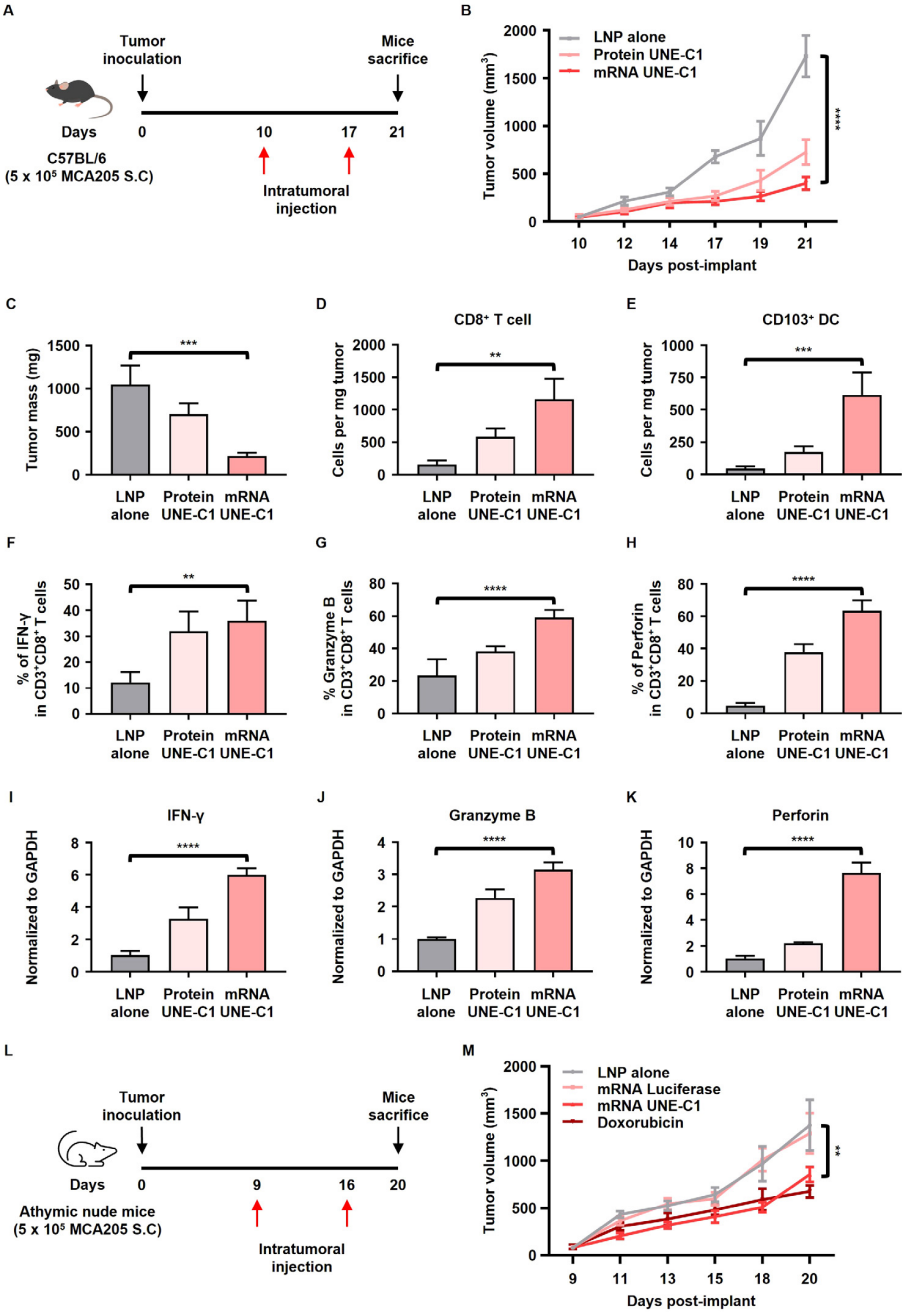
Intratumoral administration of mRNA encoding UNE-C1 induces antitumor effects in a syngeneic mouse model. **(A)** Treatment scheme for C57BL/6 mice bearing MCA205 tumors. **(B)** Tumor volume growth curve following intratumoral treatment with protein UNE-C1, mRNA UNE-C1, or lipid nanoparticle (LNP) alone. **(C)** The tumor mass collected on day 21. **(D–H)** Quantification of tumor-infiltrating **(D)** CD8^+^ T cells, **(E)** CD103^+^ DCs, **(F)** IFN- γ^+^ CD8^+^, **(G)** Granzyme B^+^ CD8^+^, and **(H)** perforin^+^ CD8^+^ T cells in MCA205-bearing mice via flow cytometry. **(I**–**K)** Relative mRNA expression levels of **(I)** IFN-γ, **(J)** Granzyme B, and **(K)** perforin in tumor tissues. **(L)** Treatment scheme for athymic nude mice bearing MCA205 tumors. **(M)** Tumor volume growth curve following intratumoral treatment with mRNA UNE-C1, mRNA luciferase, doxorubicin, or LNP alone. Data represent results from three independent experiments. Results are presented as mean ± SEM. Statistical significance was determined using one-way ANOVA (**p < 0.01, ***p < 0.001, and ****p < 0.0001).

To evaluate the antitumor efficacy of mRNA encoding UNE-C1, MCA205 tumor-bearing mice were intratumorally injected with either recombinant protein UNE-C1 (Protein UNE-C1), mRNA UNE-C1 formulated in LNP, or LNP alone (**Figure 4A**). Up to 21 d after tumor inoculation, both protein UNE-C1 and mRNA UNE-C1 significantly inhibited tumor growth compared to LNP alone (**Figures 4B, C**). Notably, the mRNA UNE-C1 group exhibited a slightly higher suppression of tumor growth than the protein UNE-C1 group. The administration of mRNA UNE-C1 led to a higher proportion of CD8^+^ T cells within the tumor microenvironment compared to the protein treatment (**Figure 4D**). Additionally, mRNA UNE-C1 significantly enhanced the proportion of migratory CD103^+^ DCs, which are crucial for eliciting an antitumor T cell response (**Figure 4E**). The levels of IFN-γ, Granzyme B, and perforin, critical mediators of cytotoxic responses, were significantly elevated in both CD8^+^ and CD4^+^ T cells (**Figures 4F–H**, **Supplementary Figures 4D–F**). Moreover, transcript levels of these associated cytokines were also increased (**Figures 4I–K**). These findings indicate that mRNA UNE-C1 effectively induces antitumor immune responses characterized by increased infiltration of CD8^+^ T cells and migratory CD103^+^ DCs within the tumor microenvironment, as well as an enhanced cytotoxic T cell response.

To confirm whether UNE-C1-induced cancer cell death occurs *in vivo*, an anticancer efficacy experiment was conducted using UNE-C1 in athymic nude mice, which lack a thymus and T cells (**Figure 4L**). The results showed that mRNA UNE-C1 slightly inhibited tumor growth, similar to the effect of doxorubicin (**Figure 4M**), demonstrating the potential of UNE-C1 to induce cell death in cancer cells *in vivo*. Moreover, the antitumor efficacy of mRNA UNE-C1 was lower in the athymic nude mice model compared to the immunocompetent model, suggesting that adaptive immune mechanisms are also involved in UNE-C1-mediated immunotherapies.

### mRNA encoding UNE-C1 induces ICD and enhances DC infiltration in the tumor microenvironment

**Figure 5:**
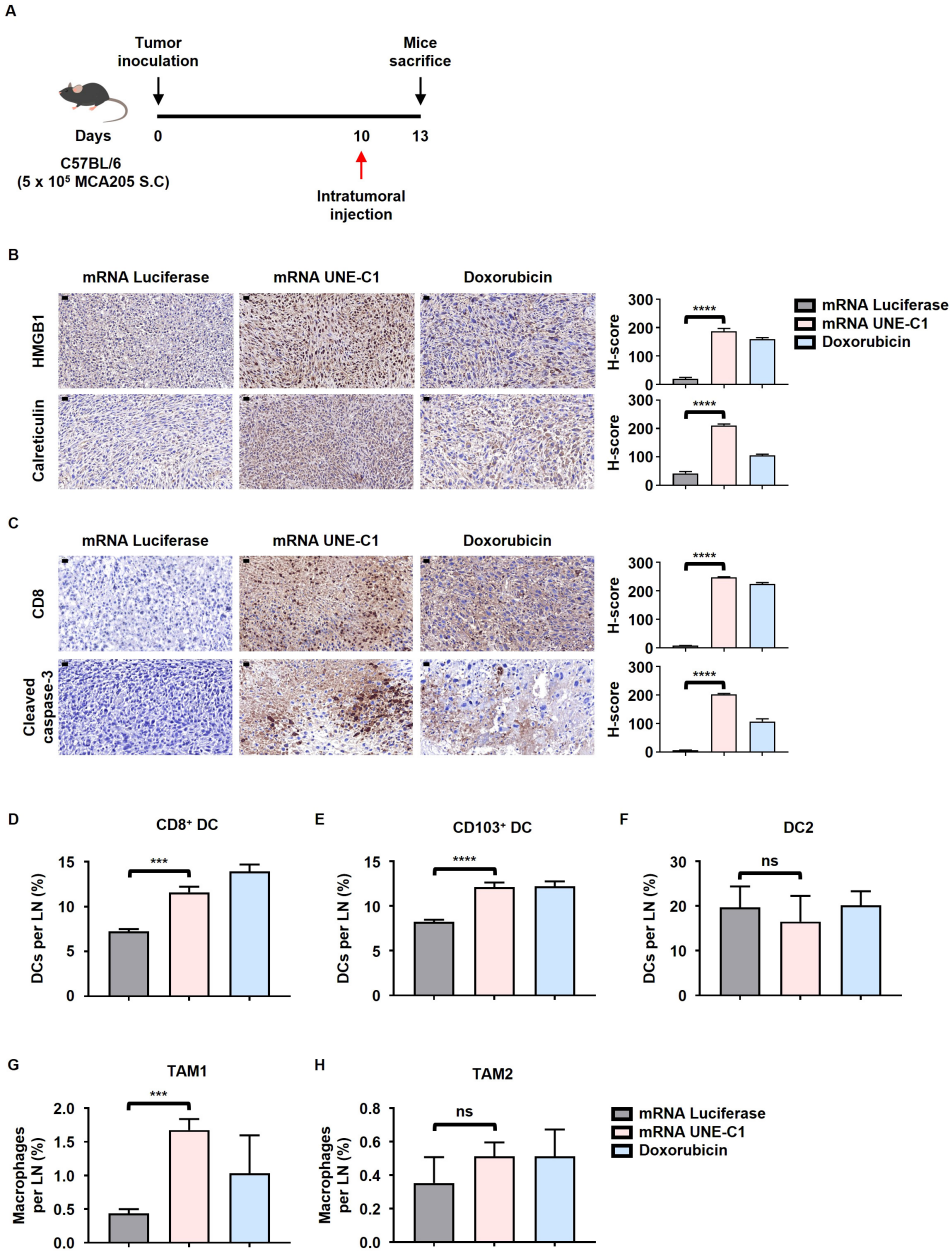
Intratumoral administration of mRNA encoding UNE-C1 induces ICD and enhances the infiltration of CD103^+^ migratory DCs and CD8^+^ resident DCs within the tumor tissue. **(A)** Treatment scheme for C57BL/6 mice bearing MCA205 tumors. **(B, C)** Representative immunohistochemistry images showing **(B)** HMGB1 and calreticulin staining, and **(C)** CD8 and cleaved caspase-3 staining, with corresponding quantification results (scale bars = 20 μm) (magnification: 40x). **(D**–**H)** Quantification of **(D)** CD8^+^, **(E)** CD103^+^ DCs, **(F)** DC2, **(G)** TAM1 and **(H)** TAM2 in TdLNs via flow cytometry. Results are presented as mean ± SD. Statistical significance was determined using one-way ANOVA (***p < 0.001, ****p < 0.0001). ns, not significant.

To further evaluate the mechanisms through which UNE-C1 mediates ICD *in vivo*, we conducted intratumoral administrations of mRNA luciferase, mRNA UNE-C1, and doxorubicin (**Figure 5A**). The delivery of mRNA luciferase and mRNA UNE-C1, which are similar in size, was accomplished using LNP (**Supplementary Figure 3A**). Both mRNA-LNP formulations exhibited comparable mRNA encapsulation efficiencies post-synthesis (**Supplementary Figure 3D**). Histological analysis revealed that mRNA UNE-C1 treatment led to elevated expression levels of HMGB1 and calreticulin compared to mRNA luciferase (**Figure 5B**). In particular, calreticulin was significantly upregulated in response to mRNA UNE-C1 compared to doxorubicin. Both mRNA UNE-C1 and doxorubicin increased the expression levels of cleaved caspase-3 (**Figure 5C**). These findings suggest that mRNA UNE-C1 induced ICD within the tumor microenvironment, indicating a specific effect rather than a non-specific response to mRNA.

Additionally, the mRNA UNE-C1 treatment group exhibited an increase in migratory CD103^+^ and lymphoid-resident CD8^+^ DCs within the Tumor-draining lymph nodes (TdLN), both of which belong to the DC1 subset and are essential for priming CD8^+^ T cells (**Figures 5D, E**). In contrast, the DC2 population showed no significant changes (**Figure 5F**). Furthermore, the proportion of tumor-associated macrophage type 1 (TAM1) was elevated following mRNA UNE-C1 treatment, while TAM2 did not demonstrate significant changes (**Figures 5G, H**). This expansion was associated with an increase in tumor-infiltrating CD8^+^ T cells in the mRNA UNE-C1 treatment group (**Figure 5C**). These findings indicate that the expansion of CD8^+^, CD103^+^ DCs and TAM1 and the induction of ICD by the mRNA UNE-C1 significantly contributed to the increase in tumor-infiltrating CD8^+^ T cells.

### TLR2 and FADD expression levels influence the sensitivity of cancer cells to UNE-C1-induced ICD

**Figure 6:**
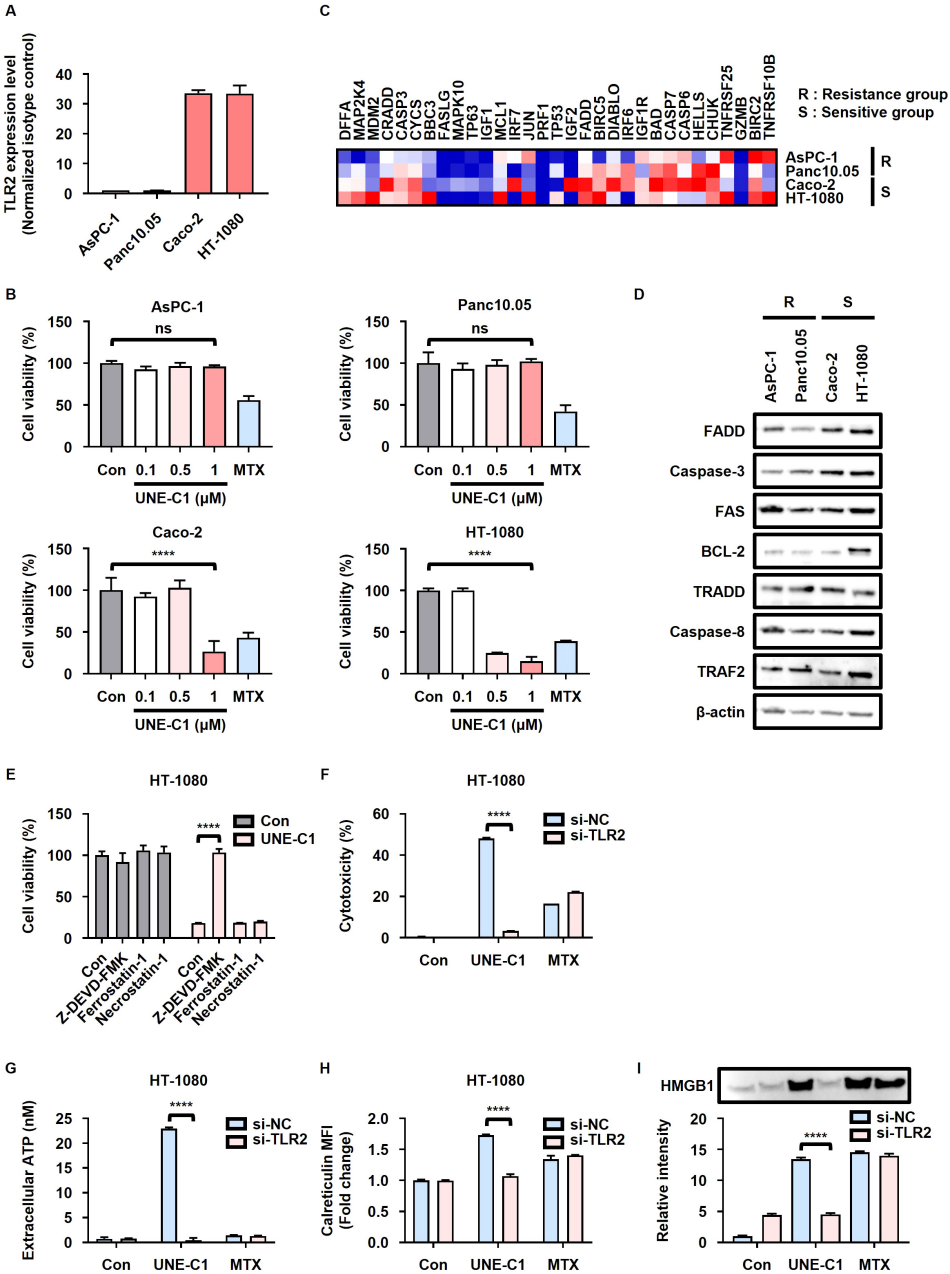
UNE-C1 elicits TLR2- and FADD-dependent ICD in human cells. **(A)** TLR2 expression in AsPC-1, Panc10.05, Caco-2, and HT-1080 cells measured using flow cytometry, presented as the ratio of the mean fluorescence intensity (MFI) of cells stained with anti-TLR2 antibody to that of cells stained with the isotype antibody. **(B)** Cell viability of AsPC-1, Panc10.05, Caco-2, and HT-1080 cells treated with UNE-C1 or MTX. **(C)** Heatmap of apoptosis-related genes between resistant and sensitive groups. Genes were ranked by scores from Gene Set Enrichment Analysis (GSEA) based on the CCLE database using the WP_apoptosis gene set. **(D)** Immunoblot analysis of FADD, caspase-3, FAS, BCL-2, TRADD, caspase-8, TRAF2, and β-actin in AsPC-1, Panc10.05, Caco-2, and HT-1080 cells. **(E)** Cell viability of HT-1080 cells pre-incubated with Z-DEVD-FMK, ferrostatin-1, or necrostatin-1, followed by treatment with UNE-C1. **(F**–**I)** Evaluation of the effect of TLR2 knockdown in HT-1080 cells in response to UNE-C1 or MTX treatment, including **(F)** assessment of cellular viability, **(G)** quantification of extracellular ATP levels, **(H)** determination of calreticulin surface expression, and **(I)** measurement of secreted HMGB1 in conditioned media. Results are presented as mean ± SD. Statistical significance was determined using one-way ANOVA (****p < 0.0001). ns, not significant.

To investigate the relationship between the sensitivity of cancer cells to UNE-C1-induced ICD and the expression levels of TLR2, we selected four distinct human cancer cell lines (AsPC-1, Panc10.05, Caco-2, and HT-1080) with varying TLR2 expression levels based on data from the Cancer Cell Line Encyclopedia (CCLE) (**Supplementary Figure 6A**). Based on TLR2 protein levels, these cell lines were categorized into low TLR2 expression (AsPC-1 and Panc10.05) and high TLR2 expression (Caco-2 and HT-1080) groups (**Figure 6A**). Cells with low TLR2 expression exhibited resistance to UNE-C1-induced ICD, while cells with high TLR2 expression were sensitive to UNE-C1-induced ICD (**Figure 6B**). Furthermore, analysis of transcriptomic data from the CCLE revealed that FADD, a critical mediator in the TLR2-induced apoptosis pathway, was enriched in cancer cells sensitive to UNE-C1 (**Figure 6C**). When comparing the protein expression levels of various cell death regulators in each cell line, the expression level of FADD was notably elevated in the sensitive group (**Figure 6D**). These results indicate that UNE-C1 induces ICD through the TLR2/FADD/caspase-3 pathway and that the expression levels of TLR2 and FADD in cancer cells are important for ICD induction by UNE-C1.

In our investigation, we revalidated UNE-C1-induced ICD in human cancer cells. Similar to our observation in MCA205 cells, the cell death initiated by UNE-C1 in Caco-2 and HT-1080 cells was impeded by the use of a caspase-3 inhibitor (**Figure 6E**; **Supplementary Figure 6B**). To confirm apoptosis via TLR2 activation, Caco-2 and HT-1080 cells were transfected with siRNA targeting TLR2, resulting in a reduction in surface TLR2 levels (**Supplementary Figures 2B, C**). Consequently, UNE-C1-induced cytotoxicity decreased considerably in cells with TLR2 knockdown (**Figure 6F**). The increases in ICD markers induced by UNE-C1, including the release of HMGB1 and ATP, as well as calreticulin exposure, were also reduced in cells with TLR2 knockdown (**Figures 6G–I**, **Supplementary Figure 6C**). Importantly, these DAMPs triggered by UNE-C1 were upregulated in a TLR2-dependent manner, unlike the effects of MTX. Overall, these outcomes strongly suggest that UNE-C1 triggers ICD in human cancer cells through a mechanism dependent on TLR2 activation, with the expression levels of TLR2 and FADD being critical determinants of this process.

## Discussion

Recent studies have demonstrated that the intratumoral administration of diverse TLR agonists holds considerable promise in combating cancer (24, 25, 32, 33). Cancer cells undergoing ICD due to intratumoral therapy significantly enhance the local immune response within the tumor microenvironment, thereby contributing to the antitumor immune response (34). This process is facilitated by the release of DAMPs and tumor antigens, which subsequently lead to increased T cell priming (35). Consequently, TLR agonists, which serve as both ICD inducers and vaccine adjuvants, are considered excellent candidates for intratumoral therapy. However, to optimize treatment outcomes, developing strategies that ensure the sustained retention of TLR ligands at the targeted local site is essential.

UNE-C1, a TLR2/6 agonist derived from CARS1, stands out as an effective anticancer vaccine adjuvant with demonstrated low toxicity compared to other TLR2/6 agonists (11). Given its favorable safety profile, UNE-C1 is an attractive candidate for intratumoral delivery with the potential for single administration efficacy against cancer. By formulating UNE-C1 in an mRNA-based delivery system, we aimed to achieve sustained localization of UNE-C1 within the tumor microenvironment. In our study, the intratumoral administration of mRNA encoding UNE-C1 via LNPs in tumor-bearing murine models resulted in the enhanced infiltration of CD103^+^ migratory DCs and CD8^+^ T cells into the tumor microenvironment (**Figures 4D, E**). This led to the activation of a cytotoxic T cell response (**Figures 4F–K**), culminating in the effective suppression of tumor growth (**Figures 4B, C**). Furthermore, mRNA UNE-C1 reprogrammed the tumor microenvironment to exhibit anti-tumorigenic properties (**Figures 5D–H**). This approach harnesses the potential of UNE-C1 as a safe and effective intratumoral therapy, demonstrating promising anticancer effects. The use of mRNA to deliver UNE-C1 ensures continuous expression and sustained efficacy, presenting an innovative and potent strategy for enhancing antitumor immune responses. By focusing on localized immune activation, this method mitigates the risk of systemic inflammation while maximizing therapeutic efficacy. Furthermore, it highlights the potential of UNE-C1 in cancer immunotherapy.

When TLR2 is stimulated, myeloid differentiation factor 88 (MyD88), an adapter protein of TLR2, interacts with the interleukin-1 receptor-associated kinases (IRAK) family, leading to the activation of NF-κB, which regulates the expression of various inflammatory cytokine genes (36). Additionally, MyD88 binds to FADD upon TLR2 stimulation, inducing apoptosis through the FADD/caspase 8 pathway (37). In immune cells, TLR2 activation induces the NF-κB pathway, leading to the inhibition of apoptosis (37). Nevertheless, in cancer cells, the presence of a similar protective mechanism to prevent apoptosis triggered by TLR2 activation appears to be absent. Previous research has demonstrated that TLR2/3 agonists induce ICD and enhance the release of DAMPs in cancer cells (26). Similarly, paclitaxel induces ICD via TLR4-dependent pathways (31). In our study, we investigated whether UNE-C1 induces ICD through TLR2 signaling in cancer cells, beyond the antigen presenting cell (APC) activation demonstrated in previous studies (11). Our results showed that UNE-C1 treatment led to ICD in cancer cells (**Figures 1A–J**). Furthermore, TLR2 knockdown prevented ICD induction by UNE-C1 (**Figures 2A–E**), and cell lines with high TLR2 expression were sensitive to UNE-C1-induced cell death (**Figures 6A, B**). These findings conclusively suggest that the TLR2 pathway plays a critical role in UNE-C1-mediated cell death and the release of immunogenic signals from cancer cells.

Previous studies have consistently shown an increase in the secretion of ARSs across various cancer types (38, 39). Research on secreted ARSs as TLR ligands has primarily focused on their paracrine effects on immune cells (10, 40–42). Given the ability of UNE-C1, when produced by cancer cells, to induce ICD in those cells, it is plausible that CARS1 may also have a similar autocrine effect. Consequently, secreted ARSs could significantly influence the immune system within the tumor microenvironment through both paracrine and autocrine mechanisms.

Our findings indicate that cancer cell lines sensitive to UNE-C1 exhibit high expression of both FADD and TLR2. Previous studies have reported increased TLR2 expression in advanced ovarian cancer patients, and abnormal FADD expression has been noted in various solid tumors, including glioma, non-small cell lung cancer (NSCLC), and hepatocellular carcinoma (HCC) (43–46). High expression levels of TLR2 and FADD in cancer cells could indicate a favorable response to treatments targeting these pathways, making them promising candidates for personalized immunotherapy strategies. Therefore, TLR2 and FADD could serve as potential biomarkers for identifying patients who might benefit from immunotherapy involving the intratumoral administration of UNE-C1 or other TLR2 agonists.

This study specifically focused on evaluating the efficacy of intratumoral UNE-C1 administration. Future research should aim to investigate combination therapies incorporating immune checkpoint inhibitors (ICIs) that are approved or in development for various cancer types. ICIs have transformed cancer treatment, providing an impactful approach to addressing solid tumors (47, 48). Despite this progress, a considerable portion of patients do not benefit from these therapies. Ineffective ICI responses can be attributed to factors such as inadequate T cell priming and immunological ignorance (49–51). Hence, it is hypothesized that agents boosting Tumor-infiltrating lymphocytes (TILs) could potentially heighten the efficacy of ICIs. Previous research on intratumoral TLR agonist delivery has shown that enhanced ICI efficacy can be achieved by stimulating immune cell recruitment and activation within the tumor microenvironment (26, 52). Thus, further studies are needed to explore the potential synergistic effects of combining UNE-C1 with ICIs in cancer treatment.

While our study employed canonical linear mRNA for UNE-C1 delivery, circular mRNA has been shown to offer more durable protein expression compared to the linear form used in this study (53). Indeed, previous research has demonstrated the anticancer effectiveness of intratumoral cytokine delivery using circular RNA (54). Although circular RNA presents challenges such as low productivity and expression efficiency, current research has been actively exploring diverse methods to address and overcome these limitations. Thus, improved circular mRNA holds promise as a platform for consistently delivering UNE-C1 to the local tumor microenvironment.

In summary, we developed an mRNA formulation encoding UNE-C1, a TLR2/6 agonist, and demonstrated its ability to induce ICD and enhance both innate and adaptive antitumor immune responses when administered intratumorally. Our study on the UNE-C1 delivery via mRNA provides an example in which mRNA encoding a TLR agonist linked to a signal peptide was introduced into cells, leading to the secretion of the TLR agonist and subsequent immune activation. This novel platform has the potential to be combined with various antigens delivered through mRNA, suggesting significant therapeutic efficacy. Furthermore, our findings revealed that cancer cells expressing high levels of TLR2 and FADD are particularly sensitive to ICD induction by UNE-C1. These results suggest that UNE-C1 could serve as a highly effective immunotherapy agent for cancer patients with elevated TLR2 and FADD expression levels.

## Data Availability

The original contributions presented in the study are included in the article/**Supplementary Materials**, further inquiries can be directed to the corresponding author/s.
