# Supplementary material for: Intratumoral delivery of mRNA encoding the endogenous TLR2/6 agonist UNE-C1 induces immunogenic cell death and enhances antitumor activity
Source: Front Immunol. 2024 Nov 28;15:1454504. doi: 10.3389/fimmu.2024.1454504 (PMC11634859; doi:10.3389/fimmu.2024.1454504)
Supplement: Supplementary file 1 [file DataSheet1.docx]

Supplementary Material


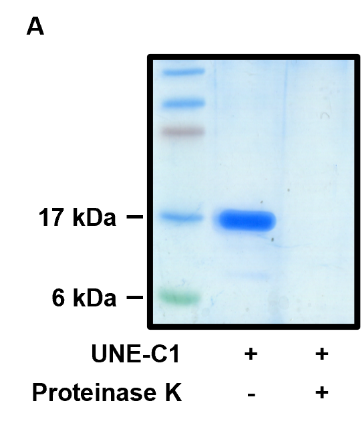


**Supplementary Figure 1.** Digestion of UNE-C1 by proteinase K. **(A)** Representative SDS-PAGE gel image showing proteinase K digestion of UNE-C1. 20 µL of UNE-C1 (1 mg/mL) samples were subjected to digestion by 100 µg/mL of proteinase K for 1 h at 37 °C and boiled for 5 min at 100 °C.


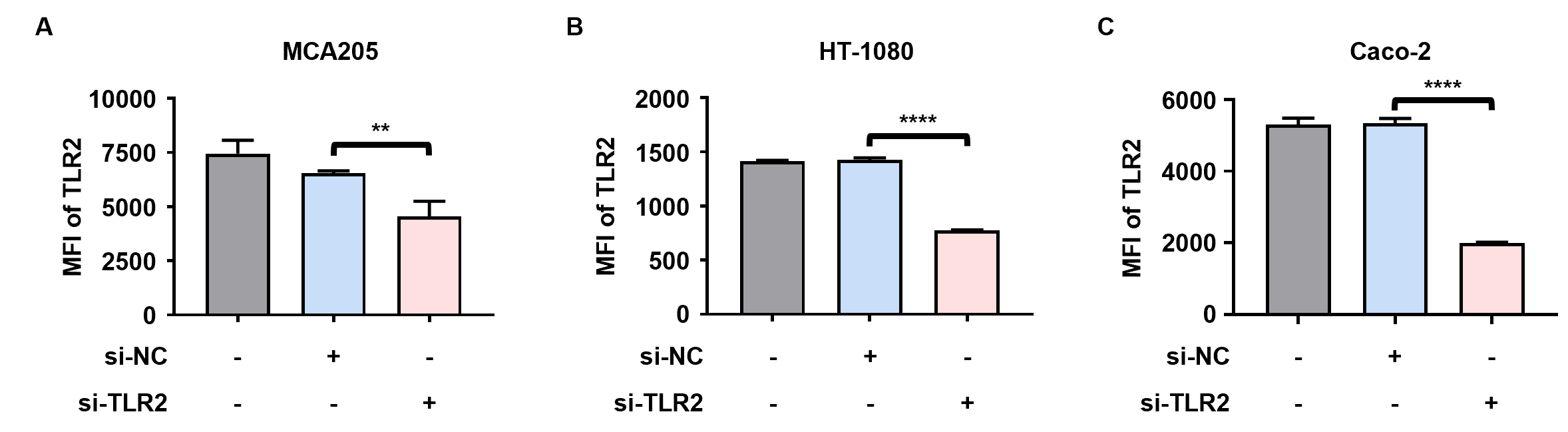


**Supplementary Figure 2.** TLR2 knockdown with siRNA in MCA205, HT-1080, and Caco-2 cells. **(A-C)** MFI was determined by flow cytometry to evaluate the surface expression of TLR2 on **(A)** MCA205, **(B)** HT-1080, and **(C)** Caco-2 cells upon transfection with negative control siRNA (si-NC) or TLR2 siRNA (si-TLR2) for 24 h. Results are presented as mean ± SD. Statistical significance was determined using one-way ANOVA (***p* < 0.01, *****p* < 0.0001).


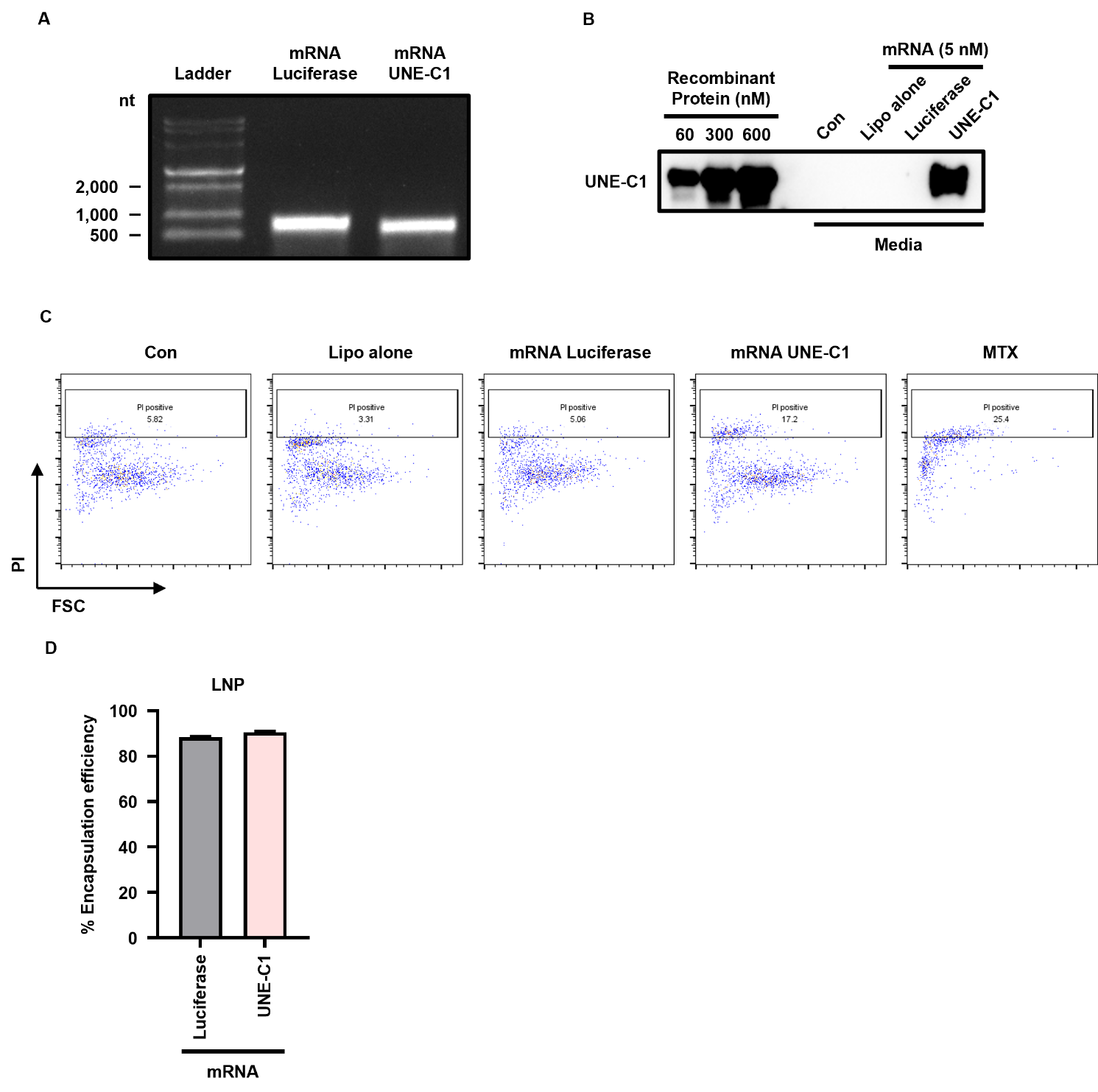


**Supplementary Figure 3.** mRNA synthesis and preparation of LNP were conducted. **(A)** Agarose gel electrophoresis was utilized to analyze the mRNA encoding either luciferase or UNE-C1. The lengths of the mRNA strands were validated through agarose gel electrophoresis performed in MOPS buffer. **(B, C)** Post-transfection with indicated mRNA or Lipofectamine alone (Lipo alone) in MCA205 cells, **(B)** immunoblot analysis for relative quantification using recombinant protein, **(C)** evaluation of cellular viability through PI staining. **(D)** The efficiency of mRNA encapsulation, determined as the ratio of encapsulated mRNA in LNP to the total mRNA, was assessed using a Quant-iT Ribogreen RNA Assay kit.


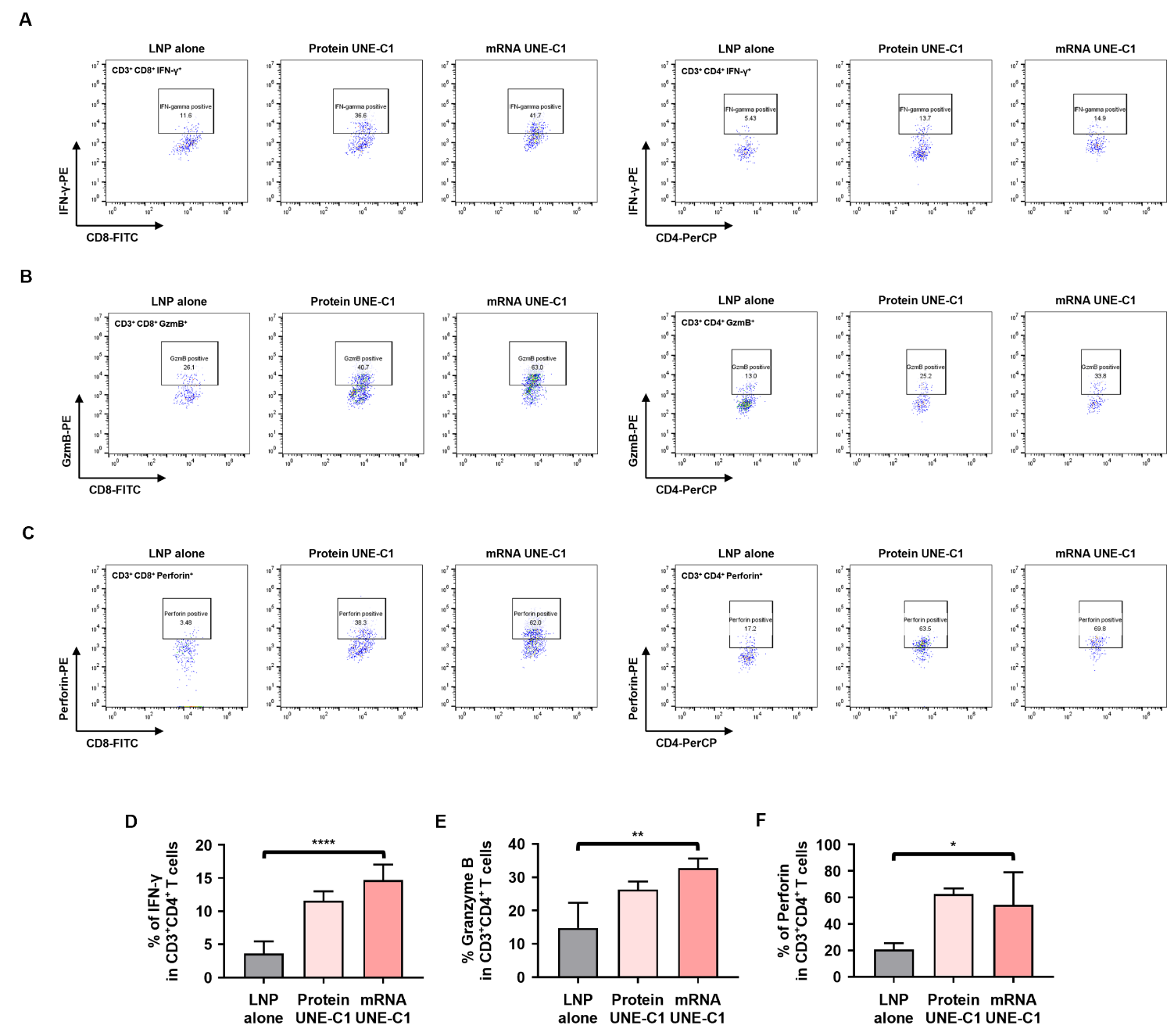


**Supplementary Figure 4.** Intratumoral administration of mRNA encoding UNE-C1 induces expression of IFN- γ, perforin, and Granzyme B in T cells. **(A-C)** Representative plots of tumor-infiltrating **(A)** IFN- γ^+^ CD8^+^, IFN- γ^+^ CD4^+^, **(B)** Granzyme B^+^ CD8^+^, Granzyme B^+^ CD4^+^, **(C)** perforin^+^ CD8^+^, and perforin^+^ CD4^+^ T cells in MCA205-bearing mice following intratumoral treatment with protein UNE-C1, mRNA UNE-C1, or LNP alone. **(D-F)** Quantification of tumor-infiltrating **(D)** IFN- γ^+^ CD4^+^, **(E)** Granzyme B^+^ CD4^+^, and **(F)** perforin^+^ CD4^+^ T cells in MCA205-bearing mice via flow cytometry. Results are presented as mean ± SD. Statistical significance was determined using two-way ANOVA (*p<0.05, ***p* < 0.01, *****p* < 0.0001).


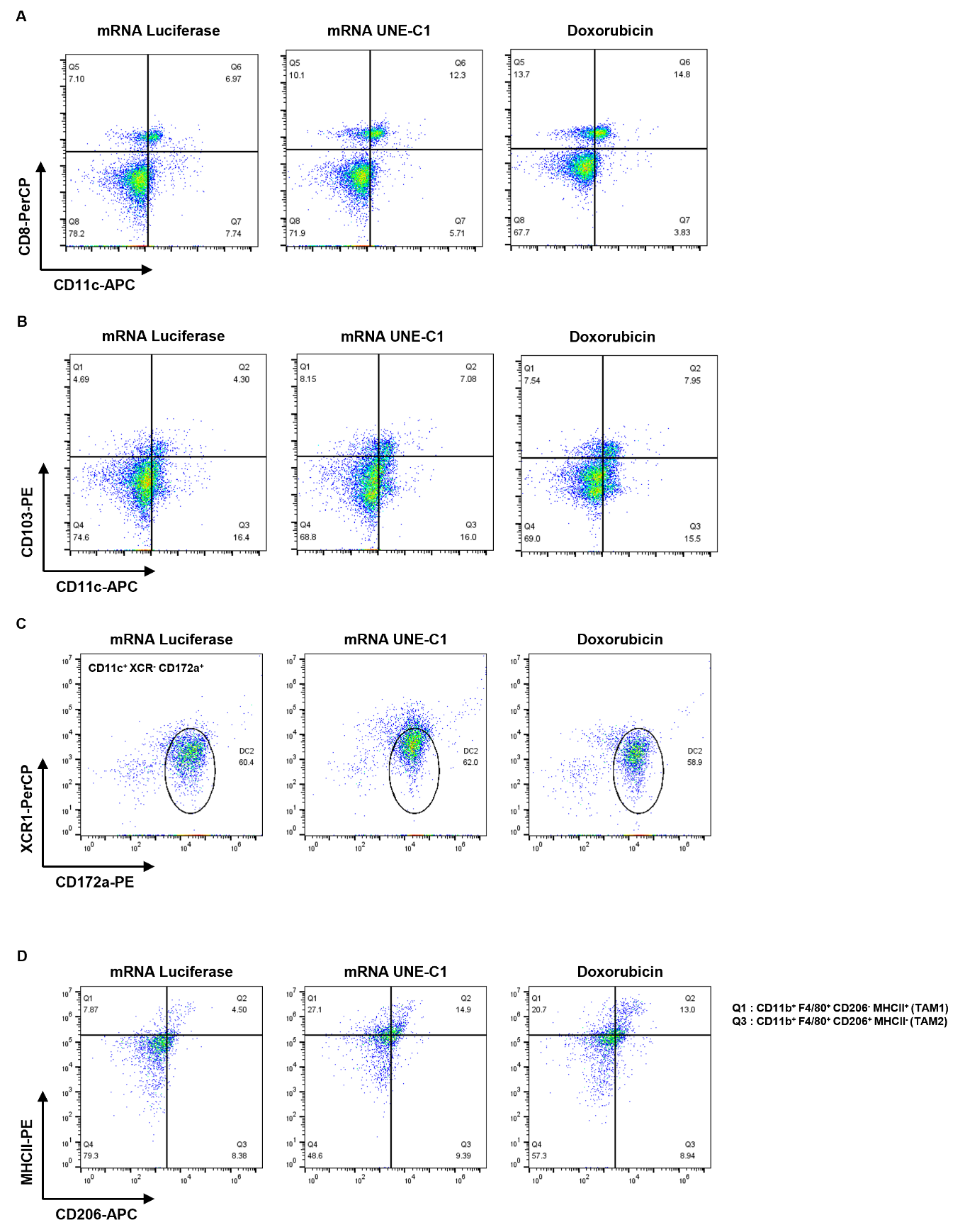


**Supplementary Figure 5.** Intratumoral administration of mRNA encoding UNE-C1 enhances the infiltration of innate immune cells within the tumor tissue. **(A-D)** Representative plots of **(A)** CD8^+^, **(B)** CD103^+^ DCs, **(C)** DC2, **(D)** TAM1 and TAM2 in TdLNs via flow cytometry.


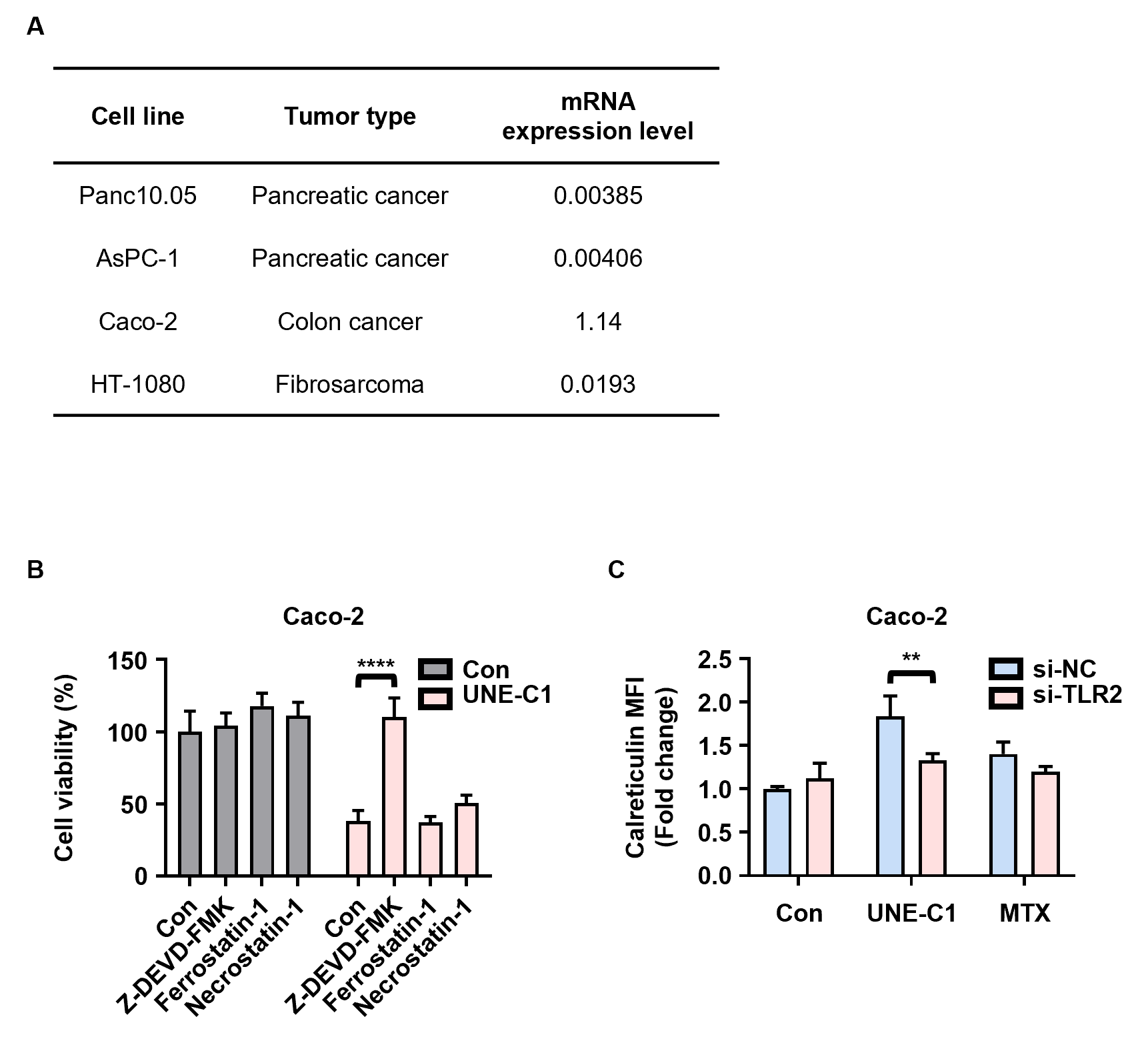


**Supplementary Figure 6.** **(A)** mRNA expression levels based on the CCLE database for four types of cancer cells. **(B)** Cell viability of Caco-2 cells treated with UNE-C1 following pre-incubation with Z-DEVD-FMK, ferrostatin-1, or necrostatin-1. **(C)** Evaluation of the effect of TLR2 knockdown in Caco-2 cells on calreticulin surface expression. Results are presented as mean ± SD. Statistical significance was determined using two-way ANOVA (***p* < 0.01, *****p* < 0.0001).
